# Posterior Circulation Endovascular Thrombectomy for Large Vessels Occlusion in Patients Presenting with NIHSS Score ≤ 10

**DOI:** 10.3390/life11121423

**Published:** 2021-12-17

**Authors:** Andrea M. Alexandre, Iacopo Valente, Arturo Consoli, Pietro Trombatore, Luca Scarcia, Mariangela Piano, Nicola Limbucci, Joseph Domenico Gabrieli, Riccardo Russo, Antonio Armando Caragliano, Maria Ruggiero, Andrea Saletti, Guido Andrea Lazzarotti, Marco Pileggi, Mirco Cosottini, Fabio Pilato, Artur Slomka, Francesca Colò, Francesca Giubbolini, Giovanni Frisullo, Giacomo Della Marca, Aldobrando Broccolini, Alessandro Pedicelli

**Affiliations:** 1 UOC Radiologia e Neuroradiologia, Dipartimento di Diagnostica per Immagini, Radioterapia Oncologica ed Ematologia, Fondazione Policlinico Universitario A. Gemelli IRCCS, 00168 Rome, Italy; iacopo.valente@policlinicogemelli.it (I.V.); alessandro.pedicelli@policlinicogemelli.it (A.P.); 2 Diagnostic and Interventional Neuroradiology, Foch Hospital, 92150 Suresnes, France; onemed21@gmail.com (A.C.); russoriccardo18@gmail.com (R.R.); 3 Università Cattolica del Sacro Cuore, 00168 Rome, Italy; pietro.tr@hotmail.it (P.T.); lucascarcia@icloud.com (L.S.); colofra94@gmail.com (F.C.); francesca.giubbolini@gmail.com (F.G.); giovanni.frisullo@policlinicogemelli.it (G.F.); giacomo.dellamarca@policlinicogemelli.it (G.D.M.); aldobrando.broccolini@policlinicogemelli.it (A.B.); 4 Neuroradiologia, ASST Grande Ospedale Metropolitano Niguarda, 20162 Milano, Italy; mariangela.piano@ospedaleniguarda.it; 5 UOC Interventistica Neurovascolare, A.O.U. Careggi, 54134 Firenze, Italy; nicolalimb@gmail.com; 6 Neuroradiology Unit, Policlinico Universitario di Padova, 35128 Padua, Italy; josephdomenico.gabrieli@aopd.veneto.it; 7 Neuroradiology Unit, Biomedical Sciences and of Morphologic and Functional Images, AOU Policlinico G. Martino, 98124 Messina, Italy; caraglia1987@gmail.com; 8 Neuroradiology Unit, AUSL Romagna, 47521 Cesena, Italy; maria.ruggiero@auslromagna.it; 9 Interventional Neuroradiology, S. Anna University Hospital of Ferrara, 44122 Ferrara, Italy; a.saletti@ospfe.it; 10 Department of Neuroradiology, Azienda Ospedaliero Universitaria Pisana (AOUP), 56126 Pisa, Italy; g.lazzarotti@ao-pisa.toscana.it (G.A.L.); mirco.cosottini@unipi.it (M.C.); 11 Department of Neuroradiology, Neurocenter of Southern Switzerland, 6900 Lugano, Switzerland; marco.pileggi@gmail.com; 12 Neurology, Neurophysiology and Neurobiology Unit, Department of Medicine, Università Campus Bio-Medico di Roma, 00128 Rome, Italy; f.pilato@unicampus.it; 13 Department of Pathophysiology, Nicolaus Copernicus University in Torun, Ludwik Rydygier Collegium Medicum, 87-100 Torun, Poland; artur.slomka@cm.umk.pl; 14 Fondazione Policlinico Universitario A.Gemelli, IRCCS, UOC Neurologia, Università Cattolica del Sacro Cuore, 00168 Roma, Italy

**Keywords:** stroke, endovascular thrombectomy, posterior circulation stroke, basilar artery, low NIHSS

## Abstract

Mechanical thrombectomy (MT) is currently the gold standard treatment for ischemic stroke due to large vessel occlusion (LVO). However, the evidence of clinical usefulness of MT in posterior circulation LVO (pc-LVO) is still doubtful compared to the anterior circulation, especially in patients with mild neurological symptoms. The database of 10 high-volume stroke centers in Europe, including a period of three year and a half, was screened for patients with an acute basilar artery occlusion or a single dominant vertebral artery occlusion (“functional” BAO) presenting with a NIHSS ≤10, and with at least 3 months follow-up. A total of 63 patients were included. Multivariate analysis demonstrated that female gender (adjusted OR 0.04; 95% CI 0–0.84; *p* = 0.04) and combined technique (adj OR 0.001; 95% CI 0–0.81; *p* = 0.04) were predictors of worse outcome. Higher pc-ASPECTS (adj OR 4.75; 95% CI 1.33–16.94; *p* = 0.02) and higher Delta NIHSS (adj OR 2.06; 95% CI 1.16–3.65; *p* = 0.01) were predictors of better outcome. Delta NIHSS was the main predictor of good outcome at 90 days in patients with posterior circulation LVO presenting with NIHSS score ≤ 10.

## 1. Introduction

Mechanical thrombectomy (MT) is currently the gold standard for the treatment of ischemic stroke due to large vessel occlusion (LVO). However, the evidence of clinical usefulness of MT in posterior circulation LVO (pc-LVO) is still doubtful compared to the anterior circulation because of the lack of specific randomized clinical trials. We assume that MT could be of great value in the treatment of posterior circulation large vessel occlusions, as it is already demonstrated in the anterior circulation.

Current guidelines in basilar artery occlusion recommend the use of recombinant tissue plasminogen activator (rtPA) within 4.5 h of symptom onset, while mechanical thrombectomy with stent retrievers can be reasonable for carefully selected patients with acute ischemic stroke in whom treatment can be initiated (groin puncture) within 6 h of symptom onset, and in very selected patients beyond 6 h from symptom onset. In both cases the class of recommendations is IIb, and the level of evidence is low (respectively C-LD: Non randomized observational studies with limitations in design or execution or Metanalysis of such studies; and B-R: nonrandomized studies). Generally, the administration of rtPA should not delay the possibility of MT [1].

One of the most relevant study on pc-LVO (BASICS study) found no significant difference in terms of functional outcome between patients undergoing MT and best medical therapy [2]. However, new devices and techniques are available and more recent studies have supported the safety and efficacy of MT for patients with pc-LVO [3].

Even more debated is the treatment of patients with pc-LVO and mild neurological symptoms (low NIHSS score). The National Institutes of Health Stroke Scale, or NIH Stroke Scale (NIHSS) is a tool used by healthcare providers to objectively quantify the impairment caused by a stroke. The NIHSS is composed of 11 items, each of which scores a specific ability between a 0 and 4. For each item, a score of 0 typically indicates normal function in that specific ability, while a higher score is indicative of some level of impairment. The individual scores from each item are summed in order to calculate a patient’s total NIHSS score. The maximum possible score is 42, with the minimum score being a 0 [4].

Several NIHSS cut-off values have been previously used to define a condition of mild neurological deterioration in patients with pc-LVO, that are usually higher than the threshold established for stroke due to anterior circulation LVO [5,6]. Indeed, a baseline NIHSS ≤ 10 has been set as the threshold for identifying patients with low/moderate neurological symptoms in previous studies [7].

Here we report a retrospective multicenter analysis of prospectively collected data on patients with acute ischemic stroke due to pc-LVO presenting with mild neurological symptoms. The aim of our study is to analyze the outcomes of patients with pc-LVO and low baseline NIHSS score (≤10) who underwent MT and to identify predictive factors of favorable outcome in this subgroup of patients, since the scientific evidence regarding this specific subgroup of patients are weak.

## 2. Materials and Methods

### 2.1. Outline of the Study and Patients’ Selection

The database of 10 high-volume stroke centers in Europe, including a period of three years and a half (2016–2019), was screened for patients with an acute basilar artery occlusion or a single dominant vertebral artery occlusion (“functional” BAO) presenting with a NIHSS ≤ 10, and with at least 3 months follow-up. Patients with incomplete follow-up data, or with a baseline National Institutes of Health Stroke Scale (NIHSS) > 10 were excluded. The following data were collected and reviewed: age, sex, administration of intravenous thrombolysis, baseline and post-procedure NIHSS score (immediately after the procedure in awake patients or when waking up from anesthesia in intubated patients), Delta NIHSS score (baseline NIHSS score—post-procedure NIHSS score), posterior circulation-Alberta Stroke Program Early CT Score (pc-ASPECT), stroke etiology, first-line thrombectomy technique, use of bail-out stenting or Percutaneous Transluminal Angioplasty (PTA), reperfusion grade (assessed using the modified Treatment in Cerebral Infarction -mTICI- scale) [8], first-passage successful recanalization (mTICI ≥ 2b), use of large-bore aspiration catheters (inner lumen > 0.060 inch), onset-to-groin time (determined as the time from stroke onset—or from time last-seen-well in cases of unwitnessed stroke—and the time of arterial puncture), procedure duration (defined as the time between arterial puncture and the final angiogram), time from onset to reperfusion (defined as the interval between the time of groin puncture and the final angiogram), procedure-related complications, post-procedural complications, and 90-day modified Rankin Scale (mRS) score. Presumed stroke etiology was derived through the angiographic assessment [9].

The present study follows the ethical recommendations of the Declaration of Helsinki. The study protocol was approved by the Ethics Committee of each participating institution. Informed consent was obtained from patients who were able to give it or, alternatively, from the legal representative of the patient. Baseline imaging included CT and/or MRI, in each case according to the imaging protocol of the involved center. Intravenous thrombolysis with recombinant tissue plasminogen activator was administered before thrombectomy in eligible patients according to current guidelines [1].

### 2.2. Endovascular Thrombectomy and Functional Outcome Assessment

All procedures were performed under general anesthesia or mild sedation depending on patient’s clinical conditions, using an 8F or 9F femoral artery approach. Direct aspiration first pass technique (ADAPT), stent retriever or a combined technique was used at the discretion of the interventional neuroradiologist. In case of reperfusion failure (mTICI 0/2a) a possible switch toward another strategy was considered (stenting and/or PTA).

The functional outcome was clinical independence, defined as mRS score 0–2 at 90-days assessed through a structured interview, either in person or by telephone, by a trained neurologist at each center.

### 2.3. Statistics

The baseline characteristics were compared between patients with favorable (mRS score 0–2) and unfavorable (mRS score 3–6) outcome at 3 months. For continuous measures, means and SD, medians and interquartile ranges (IQR) are presented and *p*-values are calculated with a two-tailed t-test for Gaussian continuous variables and the Mann-Whitney U or Kruskal-Wallis test for non-Gaussian continuous variables. Normality distribution was tested with Shapiro Wilk’s test. For categorical measures, frequencies and percentages are presented and *p*-values are calculated with a χ2 or a two-tailed Fisher’s exact test as appropriate. Multivariate analysis was performed using a logistic regression model with 90 days favorable outcome as dependent variable; except for age and sex as confounding factors, only variables with *p*-value less than 0.1 at univariate analysis were included into the multivariate model. An interaction term between center and technique used was included into both multivariable models to control for the possible effect modification by center [10]. Due to multicollinearity reasons we decided to exclude from the analysis all the variables (apart from confounding factors) included in the multivariate analysis with a variable-inflating factor (VIF) greater than 2.5. A subgroup analysis on mildly symptomatic patients was performed with 90-days favorable outcome as dependent variable. Statistical analyses were conducted using STATA 15.1 (TM, StataCorp LLC—4905 Lakeway Dr, Lakeway, TX, USA).

## 3. Results

**Figure 1:**
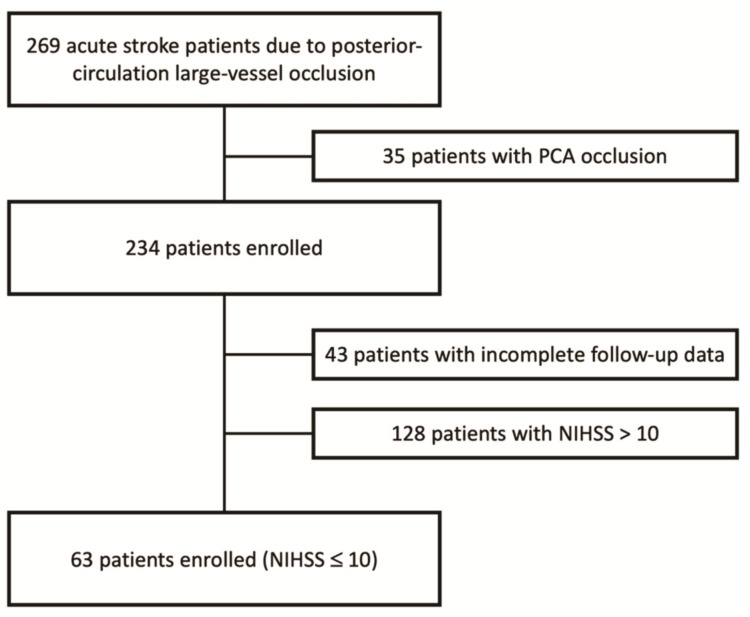
Enrollment flow-chart.

Sixty-three patients with pc-LVO and a baseline NIHSS score ≤ 10 were subjected to MT (Figure 1).

The mean age was 67.29 (63.73–70.84) and 15 patients (23.8%) were women. The rate of successful reperfusion (TICI 2b/3) was 86.4%. A favorable outcome (90 days mRS 0-2) was recorded in 37/63 patients (58.7%). Results of univariate logistic regression analysis for predicting good outcome are reported in Table 1.

The model included age, sex, administration of intravenous thrombolysis, baseline and post-procedure National Institute of Health stroke scale (NIHSS) score, Delta NIHSS, pc-ASPECT, stroke’s etiology, first-line thrombectomy technique, use of PTA, successful reperfusion (mTICI ≥ 2b), first-passage successful recanalization (mTICI ≥ 2b), use of large-bore aspiration catheters, onset-to-groin time, procedure duration, time from onset to reperfusion, procedure-related complications, and post-procedural complications. Univariate analysis showed an association between good outcome (mRS0-2) and administration of intravenous thrombolysis (*p* = 0.02), higher pc-ASPECT (mean 8.57 ± 0.53; *p* = 0.02), first-pass successful reperfusion (*p* = 0.05), and aspiration technique (*p* = 0.04). Female gender, atherosclerotic etiology, and combined technique were instead associated with poor outcome (mRS 3-6). Higher Delta NIHSS was strongly associated with a good outcome (*p* < 0.01).

Table 2 summarizes the results of multivariate logistic regression analysis for predicting factors of good outcome. The model included sex, administration of intravenous thrombolysis, presumed stroke etiology, pc-ASPECT, first-line thrombectomy technique, first-pass successful recanalization, and Delta NIHSS. Multivariate analysis confirmed female gender (adjusted OR 0.04; 95% CI 0–0.84; *p* = 0.04) and combined technique (adj OR 0.001; 95% CI 0–0.81; *p* = 0.04) as predictors of worse outcome. Higher pc-ASPECTS (adj OR 4.75; 95% CI 1.33–16.94; *p* = 0.02) and higher Delta NIHSS (adj OR 2.06; 95% CI 1.16–3.65; *p* = 0.01) were predictors of better outcome.

There were in total nine complications: four were intraprocedural, and five were post-treatment. These complications were respectively: embolization in a new territory (three cases), vessel perforation (one case); three asymptomatic subarachnoid hemorrhage, one intraparenchymal hematoma (PH) type 1, and one PH type 2 according to Heidelberg classification [11].

## 4. Discussion

Management of patients with ischemic stroke due to pc-LVO and mild neurological symptoms represents one of the most complex scenario because of its unpredictable outcome. Indeed, some of these patients may spontaneously recanalize while others will deteriorate [5]. In addition, the NIHSS score does not accurately assess the severity of the stroke in the vertebrobasilar territory [12]. As a matter of fact, the NIHSS score is heavily weighted toward hemispheric disease, and with its focus on language function, even more heavily weighted toward left hemisphere versus the right hemisphere stroke. Indeed, symptoms such as vertigo, dysphagia, gait disturbance, nystagmus, cranial nerve palsies, and ataxia are not adequately assessed by the NIHSS score [13]. On the other hand, such symptoms associated with posterior circulation stroke may lead to an under-recognition of this pathology by clinicians, as well as other less common symptoms, such as nausea and vomiting, imbalance, ill-defined dizziness, and isolated vertigo [14,15]. Physicians must consider that patients presenting at the emergency department with a low NIHSS score may have a posterior circulation stroke. Diagnosis of posterior circulation stroke may be delayed, since many patients present with a variety of signs and symptoms [16]. Therefore, the threshold for the definition of mild neurological impairment in hemispheric stroke does not seem appropriate and other authors have suggested that the NIHSS cutoff that most accurately predicts outcome is 4 points higher in anterior circulation compared to posterior circulation infarctions, suggesting a poor outcome in patients with posterior circulation strokes and low NIHSS scores [17]. Starting from these considerations, and accordingly to the BASICS study [18], we set the threshold of 10 points to identify patients with a minor stroke in the posterior circulation.

In our study, a higher Delta NIHSS was the main predictor of good outcome at 90 days in patients with posterior circulation LVO and with a baseline NIHSS score ≤ 10. Therefore, the clinical evaluation after the procedure would probably best reflect the 3 months outcome compared to the other parameters.

A higher pc-ASPECT in our analysis was associated with a good clinical outcome at 3 months too. This is reasonable since we know that pc-ASPECT detects the areas of brain tissue that are already ischemic before the treatment [19], probably reflecting the status of the collateral circulation [20]. Anyhow, the evaluation of the score is difficult with CT, due to posterior fossa beam hardening artefacts, particularly in the brainstem; these limits may cause low sensitivity and great interobserver variability [21]. On the contrary, MRI is an independent predictor of good clinical outcome prior to endovascular procedures [22], while the probability of good outcome rapidly decreases at each MRI pc-ASPECTS point drop [23].

In our study, 37 patients (59%) with pc-LVO and baseline NIHSS score ≤ 10 had a good clinical outcome after MT, whereas it was poor in the remaining 41%. These results are in line with those of Guenego et al., which reported a poor outcome at 3 months in more than 30% of patients despite mild initial symptoms and a successful endovascular recanalization [5].

Intravenous thrombolysis was associated with better clinical outcome in univariate analysis. Conflicting results have been reported when comparing bridging-therapy to MT alone, leading to several doubts about positive or negative effects of intravenous thrombolysis on MT success. One of the reasons that could probably explain this controversy could be the effect of the thrombolytic agents on the clot. Rossi et al. demonstrated that administration of thrombolytic agents significantly reduces thrombus size, releasing all the main histological components (platelet, red blood cell, and fibrin). We suggest that these modifications could make the clot more easily removable with MT [24,25].

Interestingly, atherosclerotic etiology was associated with worse clinical outcome in our univariate analysis; atherosclerotic lesions are often difficult to treat: multiple passes are usually needed and the rate of recanalization is lower compared to cardioembolic etiology [26,27]. These results were not confirmed by multivariate analysis, even if the prevalence of atherosclerotic lesions is higher in the worse outcome group.

First-pass mTICI 2b/3 failed to be a predictor of good clinical outcome in multivariate analysis. Similar results were recently confirmed by other authors [28,29,30]. Nonetheless, first-pass complete reperfusion should be the goal of every MT procedure [31], since it is linked to a better clinical outcome, a lower mortality, and fewer procedural adverse events. This is probably related to the faster procedure time and to a minor risk of endothelial vascular trauma and its resulting complications [32,33,34,35,36].

Finally, we found that a combined technique was associated with bad clinical outcome. Some authors have already demonstrated as aspiration technique is associated with shorter procedural times and improved outcomes in comparison with combined technique in pc-LVO [37]. This may be related to the faster speed of the aspiration technique and to a bias in the choice of the technique, as combined technique is usually chosen over simple aspiration in more complex cases with more difficult clots to remove.

This study provides useful data to consider when dealing with endovascular treatment of specific subgroups of patients in a real-world experience, outside the context of RCTs. Moreover, it demonstrates the utility of post-procedural clinical evaluation compared to the neurological clinical evaluation at presentation (Delta NIHSS) in predicting the clinical evolution, as a 90-day mRS.

Compared to our previous work [23] regarding posterior circulation LVO occlusion, in this study we analyzed a specific subgroup of patients with mild neurological symptoms. It is interesting to note that even with mild neurological symptoms a relatively large percentage of patients will have a bad prognosis. The most interesting data are the delta NIHSS, a parameter that more than all the others is able to predict the 3 months outcome in these patients. These aspects are still slightly analyzed in the current literature and, due to the difficulty of not treating such patients in real world activity, there are still no randomized trials on the subject. Therefore, the guidelines remain uncertain about this.

### Limitations

Our study presents several limitations: first, the design of a retrospective observational study. Hypothesize stroke’s etiology, as well as other parameters (e.g., mTICI and ASPECT score) were assessed by both the neurologist and interventional neuroradiologist without a central core-lab, or without using an automated imaging software analysis [38,39]. Consequently, the possibility of selection bias cannot be excluded. Lastly, patients’ selection was not based upon a rigid stroke imaging protocol.

The efficacy of MT in patients with AIS due to basilar artery occlusion and mild neurological symptoms at presentation has been insufficiently studied in the main randomized clinical trials. Our experience is in favor of a potential benefit regarding 3 months mRS. Anyway, this issue remains open for clarification and the need for an unambiguous recommendation in clinical practice is urgent [40,41].

## 5. Conclusions

In our study, we included 63 patients presenting with basilar artery occlusion or single vertebral artery (“functional” BAO) and mild neurological impairment, treated with mechanical thrombectomy. We found that a higher Delta NIHSS was the main predictor of good outcome at 90 days in patients with posterior circulation LVO presenting with NIHSS score ≤ 10. A higher pc-ASPECT was also associated with good clinical outcome at 3 months. Combined technique and female sex were predictors of bad clinical outcome in our retrospective series.

## Figures and Tables

**Table 1:** Univariate 90-days mRS.

Variables	mRS 3–6 (*n* = 26)	mRS 0–2 (*n* = 37)	Total (63)	*p*-Value
Age (years)	66.85 (61.03–72.66)	67.59 (63.06–72.13)	67.29 (63.73–70.84)	0.84
Female	10 (38.46%)	5 (13.51%)	15 (51.98%)	0.02
rTPA	3 (11.54%)	15 (40.54%)	18 (52.08%)	0.01
Atherosclerotic	14 (53.85%)	8 (21.62%)	22 (75.47%)	0.01
pc-ASPECT (0–10)	7.54 (6.90–8.18)	8.57 (8.04–9.09)	8.14 (7.72–8.56)	0.02
First Technique				0.04
Aspiration	10 (38.46%)	27 (72.97%)	37 (58.73%)	
Stentriever	3 (11.54%)	4 (10.81%)	7 (11.11%)	
Combined	10 (38.46%)	4 (10.81%)	14 (22.22%)	
Other	2 (7.69%)	2 (5.41%)	4 (6.35%)	
PTA	2 (7.69%)	3 (8.11%)	5 (15.80%)	0.95
Wake-up-stroke	1 (3.85%)	2 (5.41%)	3 (9.25%)	0.77
mTICI 2b/3	22 (84.62%)	35 (94.59%)	57 (179.21%)	0.18
Intraprocedural Complications	3 (11.54%)	1 (2.70%)	4 (14.24%)	0.16
Post-treatment Complications	2 (7.69%)	3 (8.33%)	5 (16.03%)	0.93
Symptoms-to-Groin (minutes)	413.60 (292.21–534.99)	439.46 (335.59–543.33)	429.03 (350.62–507.44)	0.75
Symptoms-to-Reperfusion (minutes)	515.44 (350.79–680.09)	497.86 (390.96–604.75)	504.25 (414.74–593.76)	0.85
Groin-to-Reperfusion (minutes)	156.69 (1.92–311.46)	62.53 (48.76–76.30)	95.28 (40.16–150.40)	0.10
Firstpass 2b3	11 (42.31%)	25 (67.57%)	36 (109.88%)	0.05
Large-bore Catheters	5 (19.23%)	14 (37.84%)	19 (57.07%)	0.11
NIHSS Differential	−8.23 (−14.34–−2.11)	2.68 (1.73–3.63)	−1.39 (−4.09–1.31)	0.00

*p*-values by *t*-test for continuous variables and Chi2 test for binary/categorical variables.

**Table 2:** Multivariate logistic regression analysis: 90-days mRS.

mRS02	OR	*p*-Value	[95% Conf	Interval]	Sig
Female	0.04	0.04	0	0.84	*
rTPA	2.71	0.54	0.11	66.88	
Atherosclerotic	0.49	0.58	0.04	6.39	
pc-ASPECT	4.75	0.02	1.33	16.94	*
First Technique (ref. Stentriever)					
Aspiration	1.72	0.77	0.04	67.84	
Combined	0.001	0.04	0	0.81	*
Other	0.87	0.95	0.01	51.48	
Firstpass 2b3	0.35	0.44	0.02	5.01	
NIHSS Differential	2.06	0.01	1.16	3.65	*

## Data Availability

Data supporting reported results are available upon request.
